# Cytotoxic activity of NN-32 toxin from Indian spectacled cobra venom on human breast cancer cell lines

**DOI:** 10.1186/s12906-017-2018-3

**Published:** 2017-11-28

**Authors:** Saurabh S. Attarde, Sangeeta V. Pandit

**Affiliations:** Department of Zoology, Savitribai Phule Pune University, Ganeshkhind, Pune, Maharashtra 411007 India

**Keywords:** NN-32, *Naja naja*, Cytotoxity, MCF-7, MDA-MB-231, Breast cancer

## Abstract

**Background:**

Breast cancer is the most common cancer which causes significant morbidity and mortality among women worldwide. Lack of medical facilities for early detection, therapeutic strategies for treatment and side effects due to pharmacological compounds have encompassed the need for new therapies mostly from natural sources. A lot of components have been identified from different snake venoms as therapeutic agents. A group of polypeptides (60–70 amino acid residues) called cytotoxins or cardiotoxins present in an elapid family of snakes have a wide variety of pharmaceutical actions and have the tendency to damage a wide variety of cells including cancerous cells. The aim of the present study was to evaluate the cytotoxic effect of NN-32 protein toxin purified from Indian Spectacled Cobra venom against human breast cancer cell lines (MCF-7 and MDA-MB-231).

**Methods:**

The NN-32 toxin was purified by ion exchange chromatography and further by RP-HPLC. The potential anticancer effects of the NN-32 toxin on MCF-7 and MDA-MB-231 cells were evaluated using MTT, anti-proliferation, neutral red (NR) uptake and Lactate Dehydrogenase (LDH) release assay.

**Results:**

The ion exchange chromatography showed various peaks among fraction no. 35 showing cytotoxic activity and this fraction showed a single peak with retention time 3.6 mins by HPLC using C18 column. The NN-32 toxin induced cytotoxicity in MCF-7 and MDA-MB-231 cells with the IC_50_ value of 2.5 and 6.7 μg/ml respectively. The NN-32 showed significant cytotoxicity to both the cell lines along with low cytotoxicity to MCF-10A (normal breast epithelial) cells. The cytotoxic effect was further confirmed by the anti-proliferative, NR uptake and LDH release assays.

**Conclusion:**

The purified toxin NN-32 from *Naja naja* venom showed cytotoxic activity against MCF-7 (ER+) and MDA-MB-231(ER-) cells in both dose dependent and time dependent manner.

## Background

Breast cancer is the most common cancer which causes significant morbidity and mortality among women worldwide [1]. In India, over 80,000 new cases of breast cancer diagnosed and metastasis is one of the leading cause of death [2]. In recent years, due to lack of medical facilities for early detection, therapeutic strategies for treatment and side effects due to pharmacological compounds have encompassed the need for new therapies mostly from natural sources for long-term cancer prevention and treatment [3–5].

Snake venom is a rich source of many proteins, peptides, macromolecules and many cytotoxins, neurotoxins, cardiotoxins, myotoxins, dendrotoxins, haemotoxins, fibrinolytic enzymes, Phospholipase A2 etc. [6]. Calmette et al. [7] in 1933 first reported that snake venom has anticancer activity and thereafter a lot of components from different snake venoms have been identified as showing therapeutic properties. A group of polypeptides (60–70 amino acid residues) called cytotoxins or cardiotoxins present in an elapid family of snakes have a wide variety of pharmaceutical actions and have the tendency to damage a wide variety of cells including cancerous cells [8, 9].

A number of compounds from venomous animals, such as snakes, scorpions, spiders, toads, frogs, bees, caterpillars, insects, wasps, centipedes and ants have been isolated and showed therapeutic applications [10–13]. For example, a compound TM601, modified form of the peptide Chlorotoxin (CTX) purified from *Leiurus quinquestriatus* scorpion venom, has been shown to block chloride channel specifically on glioma cell surface and also shown potent pleiotropic anti-angiogenic effect, which is currently under phase-II clinical trials [14, 15].

NN-32 is a 6.7 KDa proteinous toxin isolated from Indian spectacled cobra venom, which showed antioxidant and antitumor properties against EAC bearing BALB/c mice [16]. The present study reports the cytotoxic activity of a toxin NN-32 present in *Naja naja* venom against human breast cancer cell lines.

## Methods

### Chemicals

Carboxymethyl cellulose (CM Cellulose), Dulbecco’s modified eagle medium (DMEM), Fetal bovine serum (FBS), Penicillin, Streptomycin and Trypsin-EDTA Solution were purchased from Himedia (India). Thiazolyl Blue Tetrazolium Bromide (MTT), Dimethyl Sulfoxide (DMSO), Neural Red (NR), Formaldehyde, β-Nicotinamide adenine dinucleotide reduced dipotassium salt (β-NADH), Trypan Blue and Triton X-100 dye solution were purchased from Sigma-Aldrich (USA).

### Snake venom collection and ethics approval

Lyophilized *Naja naja* [17] venom was purchased from Calcutta Snake Park, Kolkata, India and stored at 4 °C till further use. Venom concentration was expressed in terms of dry weight/protein equivalent. The study protocol was approved by the Institutional Biosafety Committee (IBSC) of Savitribai Phule Pune University, Pune, India.

### Purification of NN-32

A 250 mg of Lyophilized whole venom of *Naja naja* was dissolved in 5 ml of de-ionized water and given a heat treatment for 30 min at 60^o^c in a water bath followed by centrifugation at 2500 rpm for 20 min. 50 mg of the supernatant was loaded onto a CM-cellulose column (100 × 20 mm) which was equilibrated with 0.02 M phosphate buffer (pH 7.2). A total of 42 fractions (each of 5 ml volume) were collected using the stepwise gradient of sodium chloride (0.02 M – 1 M in phosphate buffer, pH 7.2) with a constant elution rate of 30 ml/min at room temperature. Protein content in the fractions was estimated by Lawry’s method [18]. All the fractions were checked for their cytotoxic activity against MCF-7 cells. The fraction which was showing cytotoxic activity was further purified by Reverse phase HPLC (Shimadzu LC-2010HT, Japan) using C18 column (4.6 × 250 mm) (Waters, USA) equilibrated with 0.1% Trifluoroacetic acid (TFA) in water and eluted with a linear gradient of 100% acetonitrile in 0.1% TFA at a flow rate of 1 ml/min. The HPLC profile of the fraction was monitored at 280 nm for 60 min using Shimadzu Prominence UV/Vis detector (SPD-20A).

### Characterization of NN-32

MALDI-MS (Applied Biosystems, 4700 Proteomics Analyzer 170) was performed to determine the mass of the fraction protein. Mass spectrometric spectra were obtained using MALDI-TOF system. MS/MS spectra were searched using the Mascot database search engine against the NCBInr protein database.

### Cell culture

Human Breast cancer cell lines (MCF-7 and MDA-MB-231) along with Human normal breast epithelial cell line (MCF-10A) were purchased from National Facility for Animal Tissue and Cell Culture, Pune, India. They were cultured in DMEM supplemented with 10% heat-inactivated FBS, penicillin (100 units/ml) and streptomycin (10 mg/ml). Cells were grown to sub confluence at 37 °C in a humidified atmosphere of 5% CO_2_.

### MTT assay

MCF-7, MDA-MB-231 and MCF-10A cells were seeded at densities of 1 × 10^4^/well into 96-well plates and incubated at 37 °C for 24 h under 5% CO_2_. The cells were then treated with different concentrations of NN-32 (0.125–16 μg/ml) and doxorubicin (0.5–5 μM) as the positive control. After 48 h of incubation, 20 μl of MTT solution (5 mg/ml) was added into each well and incubated for 4 h. The medium was discarded and the formazan precipitate was dissolved in DMSO. The absorbance of the mixtures was determined using a microtiter plate reader at 570 nm and the cell viability expressed as percentage inhibition relative to controls. All experiments were performed in triplicates. The IC_50_ was generated for each cell line from the dose response curve.

### Anti-proliferation assay

MCF-7 and MDA-MB-231 cells were seeded at densities of 1 × 10^4^ cells/well into 6-well plates and allowed to incubate for cell attachment for 24 h. These cells were then exposed to 5, 10 and 15 μg/ml concentrations of NN-32 and the plates incubated at 37 °C under 5% CO_2_, for 24, 48, and 72 h. At the end of the incubation periods, the medium was removed and washed with cold PBS followed by the addition of 1 ml of 0.05% trypsin-EDTA. The plates were then incubated for 15 min at 37 °C and after the majority of the cells had detached from the plate, they were harvested by spinning the suspension for 10 min at 1000 rpm using Eppendorf Centrifuge 5810 R (Hamburg, Germany) and the supernatant was discarded. 20 μl of the cell pellet were re suspended in 20 μl of 0.4% trypan blue solution. The dye-excluding viable cells were counted microscopically using a haemocytometer and expressed as percent of control cells that were still viable.

### Neutral red uptake assay

The cells were seeded in 96-well plates and incubated overnight under 5% CO_2_ at 37 °C until they reached 60% confluence. The medium was then discarded and replaced with 200 μl of fresh growth medium containing the same concentrations of the NN-32 as that used in the MTT assay. Untreated cells under the same conditions were used as controls. The plates were incubated at 37 °C for 24, 48 and 72 h under 5% CO2 and the cells were then washed three times with 200 μl of PBS. The plates were further incubated at 25 °C for 3 h in medium containing 200 μl NR solutions, and the cells subsequently washed to remove the NR solution. Cells were then exposed to fixing solution consisting of 1% CaCl_2_ and 0.5% formaldehyde in milli-Q water for 2 min followed by two washes with 1% acetic acid and 50% ethanol in milli-Q water. The plates were incubated for 10 min and then read in a micro plate reader at 540 nm [19].

### Lactate dehydrogenase release assay

The cells were seeded in 96-well plates in 100 μl of media and then treated with different concentrations of NN-32 (0.125–16 μg/mL). The permeability of the cell membrane of MCF-7 and MDA-MB-231 cell lines after treatment with NN-32 was determined by LDH release assay [19].

### Statistical analysis

For the number of experiments indicated, data are shown as mean ± SD. The paired Student’s t test was performed to evaluate two independent groups of samples. In all analyses, *p* < 0.05 was taken as statistically significant.

## Results

### Purification of NN-32

**Fig. 1:**
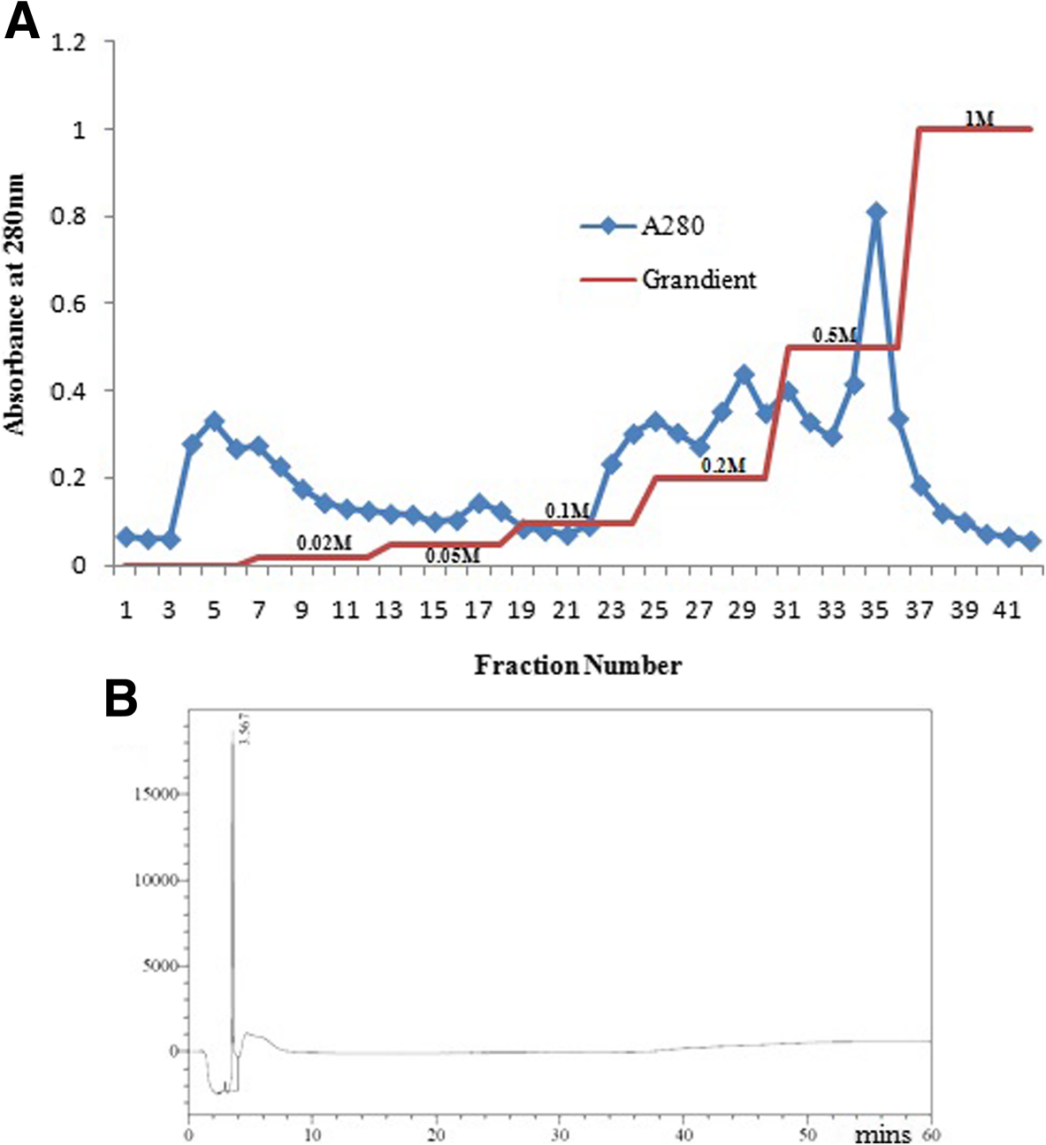
**a** Purification of NN-32. An Ion exchange chromatography of Indian spectacled cobra (*Naja naja*) venom. **b** Peak eluted with 0.5 M NaCl solution was further purified by RP-HPLC. HPLC profile showed a single peak of NN-32 with a retention time of 3.6 min

50 mg of venom applied on Ion exchange chromatography column (100 × 20 mm) resolve into several peaks (Fig. 1a). Among all the fractions, fraction number 35 was eluted with 0.5 M NaCl showed cytotoxicity against MCF-7 cell line. This fraction was further resolved through RP-HPLC using C18 column (4.6 × 250 mm), produced a single peak with a retention time of 3.6 mins (Fig. 1b).

### Characterization of NN-32

**Fig. 2:**
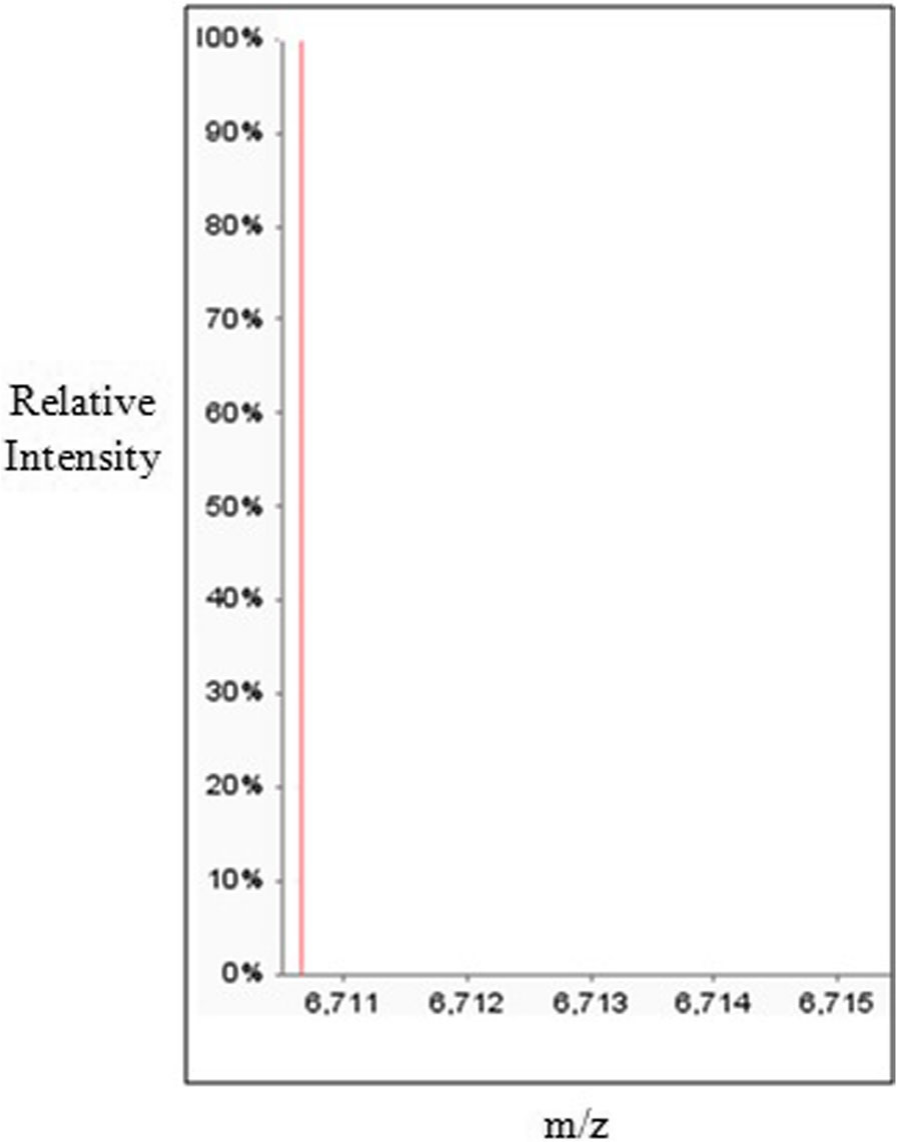
MALDI-MS spectra of NN-32 showing a molecular mass of 6.7 kDa

The molecular mass of NN-32 determined by MALDI-MS was found to be 6.7 kDa (Fig. 2). MS/MS spectra of NN-32 were searched against the NCBInr protein database using the Mascot database search engine showed the list of toxins having homology with NN-32 (Table 1).

**Table 1:** List of toxins having homology with the NN-32 toxin, search carried out by mascot database search engine against the NCBInr protein database

Sr. No	Protein ID	Protein Discription	MW. KDa
1	3SA2_NAJNA	Cytotoxin 2 OS-*Naja naja* PE = 1 SV = 2	6.75
2	3SA2A_NAJNA	Cytotoxin 2a OS = *Naja naja* PE = 1 SV = 2	6.71
3	3SA1_NAJKA	Cytotoxin 1 OS -*Naja kaouthia* PE = 1 SV = 1	6.81
4	3SA7A_NAJKA	Cytotoxin 2 OS -*Naja kaouthia* PE = 1 SV = 1	6.75
5	3SA3 NAJNA	Cytotoxin 3 OS -*Naja naja* PE = 1 SV = 1	6.75
6	3SA3_NAJKA	Cytotoxin 3 OS = *Naja kaouthia* PE = 1 SV = 1	6.72
7	3SA6_NAJSP	Cytotoxin 6 (Fragment) OS -*Naja sputatrix* PE = 3 SV = 1	7.03
8	A0A0U5ARS4_NAJNA	Cytotoxin 11 (Fragment) OS = *Naja naja* GN = CTX11 PE = 2 SV = 1	7.96
9	AO A0U4N 5 W4_NAJN A	Cytotoxin 13 (Fragment) OS-*Naja naja* GN = CTX13 PE = 2 SV = 1	7.95
10	3SA0_NAJSP	Cytotoxin OS -*Naja sputatrix* PE = 3 SV = 1	9.10
11	3SA3_NAJAT	Cytotoxin 3 OS = *Naja atra* PE = 1 SV = 1	9.04
12	3SA3_NAJSP	Cytotoxin 3 OS = *Naja sputatrix* PE = 1 SV = 1	9.04
13	3SA4_NAJKA	Cytotoxin 4 OS-*Naja kaouthia* PE = 1 SV = 1	9.04
14	3SA3A_NAJAT	Cytotoxin 3a OS = *Naja atra* PE = 3 SV = 1	9.07
15	3SA3B_NAJAT	Cytotoxin 3b OS -*Naja atra* PE = 3 SV = 2	9.02
16	3SA1D_NAJAT	Cytotoxin ld/le OS = *Naja atra* PE = 3 SV = 1	8.99
17	E2IU04_NAJAT	Three-finger toxin (Fragment) OS -*Naja atra* PE = 3 SV = 1	6.97
18	Q9PS33_NAJOX	VC-l = CYTOTOXIN OS = *Naja oxiatut* PE = 3 SV = 1	6.72
19	3SAl_NAJOX	Cytotoxin 1 OS-*Naja oxiatut* PE = 1 SV = 1	6.82
20	3SA1_NAJAT	Cytotoxin 1 OS-*Naja atra* PE = 1 SV = 1	8.99
21	3SA8_NAJKA	Cytotoxin 1 OS-*Naja kaouthia* PE = 1 SV = 1	6.70
22	3SA1C NAJAT	Cytotoxin lc OS-*Naja atra* PE = 3 SV = 1	9.02
23	3SAPF_NAJAT	Cytotoxin I-like P-15 OS = *Naja atra* PE = 3 SV = 1	8.91
24	3SATF_NAJAT	Cytotoxin I-like T-15 OS-*Naja atra* PE = 3 SV = 1	9.00
25	3SA1A_NAJAT	Cytotoxin la OS-*Naja atra* PE = 3 SV = 1	8.98
26	3SA1D_NAJAT	Isoform 2 of Cytotoxin ld/le OS-*Naja atra*	10.77
27	3SA8B_NAJAT	Cytotoxin 8 OS-*Naja atra* PE = 3 SV = 1	8.90
28	3SA3D_NAJAT	Cytotoxin 3d OS -*Naja atra* PE = 3 S V = 1	8.93
29	3SAFA_NAJAT	Cytotoxin SP15a OS = *Naja atra* PE = 1 SV = 1	6.68
30	E2ITZ7_NAJAT	Three-finger toxin OS -*Naja atra* PE = 3 SV = 1	8.83

### MTT assay

**Fig. 3:**
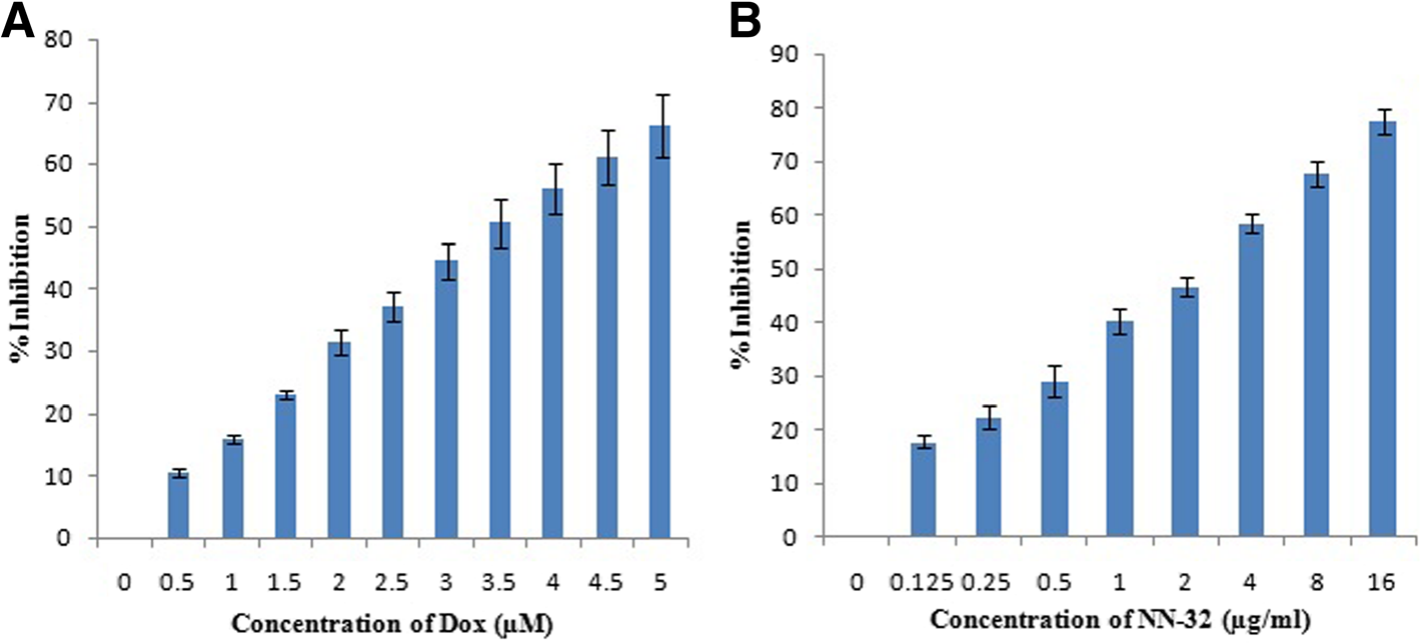
Inhibition of MCF-7 cells after 48 h of treatment with (**a**) Doxorubicin and (**b**) NN-32 toxin. Values are expressed as mean ± Std. Dev (*n* = 3)

**Fig. 4:**
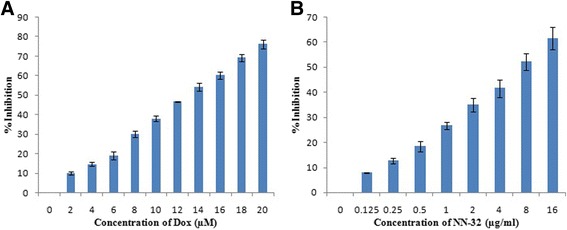
Inhibition of MDA-MB-231 cells after 48 h of treatment with (**a**) Doxorubicin and (**b**) NN-32 toxin. Values are expressed as mean ± Std. Dev (*n* = 3)

**Fig. 5:**
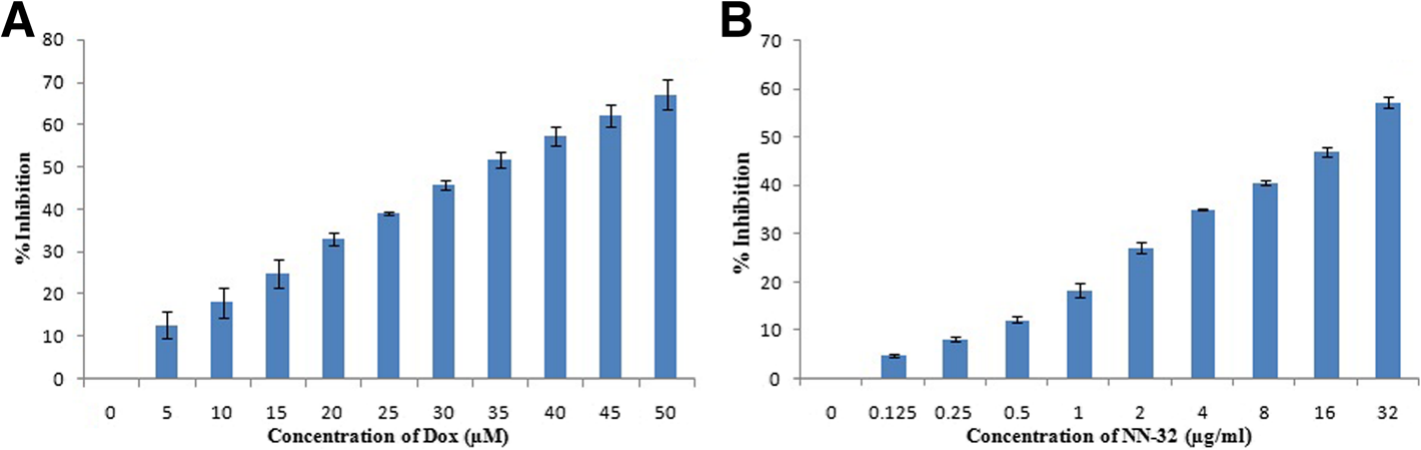
Inhibition of MCF-10A cells after 48 h of treatment with (**a**) Doxorubicin and (**b**) NN-32 toxin. Values are expressed as mean ± Std. Dev (*n* = 3)

Cytotoxic activity of NN-32 toxin was determined against two human breast cancer cell lines of which, one is estrogen receptor positive (ER+) MCF-7 cells and another is estrogen receptor negative (ER-) MDA-MB-231 cells. The percent inhibition of growth of these two breast cancer cells after treatment with the NN-32 toxin and Doxorubicin after 48 h was determined. The response of MCF-7 and MDA-MB-231 cells to increasing concentration of NN-32 toxin and Doxorubicin are shown in Figs. 3 and 4 respectively. The results showed the tendency of both cell lines to inhibit sharply upon treatment with low NN-32 and Doxorubicin concentrations, with narrowing response intensity as the concentration of NN-32 and Doxorubicin were increased. The IC_50_ values of Doxorubicin after 48 h of treatment was found out to be 4.1 μM and 15.1 μM for MCF-7 and MDA-MB-231 cell lines, while IC_50_ values of NN-32 toxin after 48 h treatment ranged between 2.5 and 6.7 μg/ml for MCF-7 and MDA-MB-231 cell lines respectively. The IC_50_ value of Doxorubicin and NN-32 after 48 h of treatment was found out to be 39.6 μM and 25 μg/ml respectively for the normal MCF-10A cells, is 10 times higher than that of the MCF-7 cells (Fig. 5). The NN-32 showed lower IC_50_ values for the cancer cells compared to the normal breast cells, suggesting that the toxin could have great potentials as an anti-cancer agent.

### Anti-proliferation assay

**Fig. 6:**
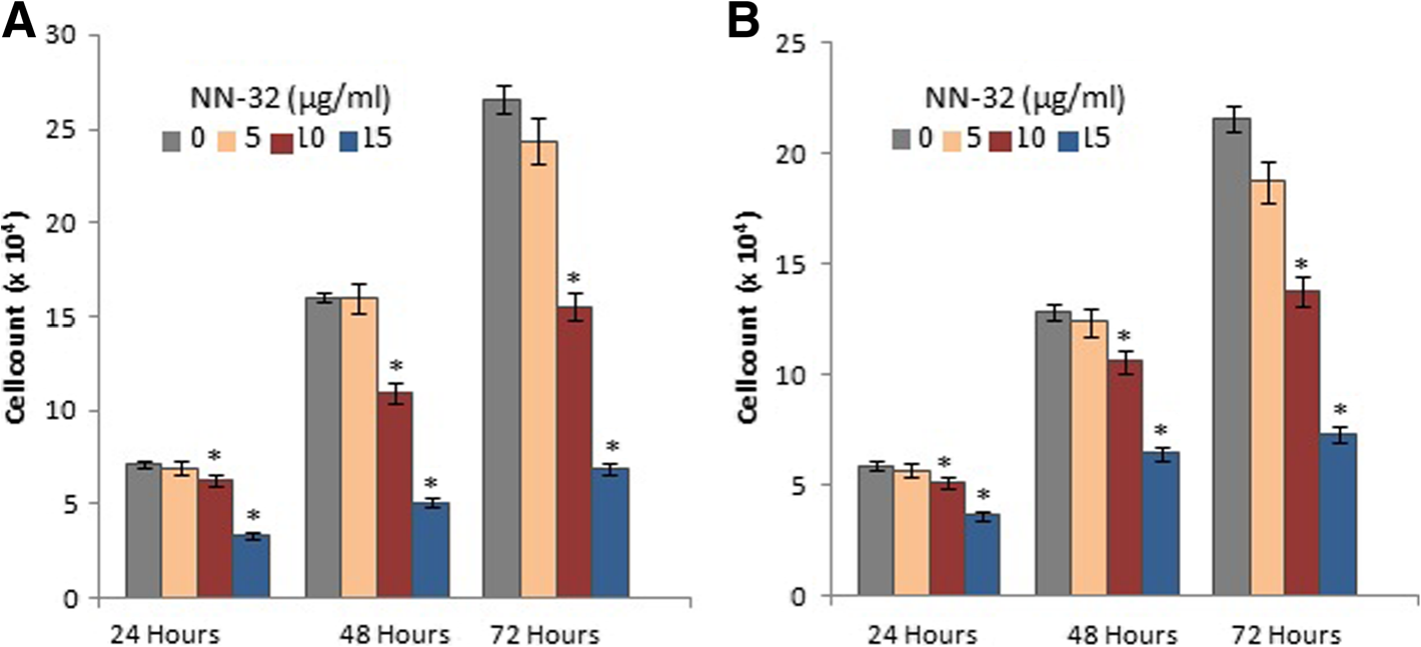
Viability of Human breast cancer cells treated with NN-32 after 24, 48 and 72 h, (**a**) MCF-7 cell and (**b**) MDA-MB-231 cell. Values are expressed as mean ± Std. Dev (*n* = 3). *p < 0.05 in comparison with the control group

The Anti-proliferative effects of the NN-32 toxin on MCF-7 and MDA-MB-231 cells were illustrated in Fig. 6. The percent survival of the MCF-7 cells after 24, 48 and 72 h of incubation with 10 μg/ml of NN-32 were 89, 68 and 58% respectively. However, MDA-MB-231 cells similarly treated with the NN-32 did not show much reduction in viability as the MCF-7 cells with values 87, 83 and 64% respectively. This suggested that the NN-32 could have a greater effect on the viability of MCF-7 than MDA-MB-231 cells.

### NR uptake assay

**Fig. 7:**
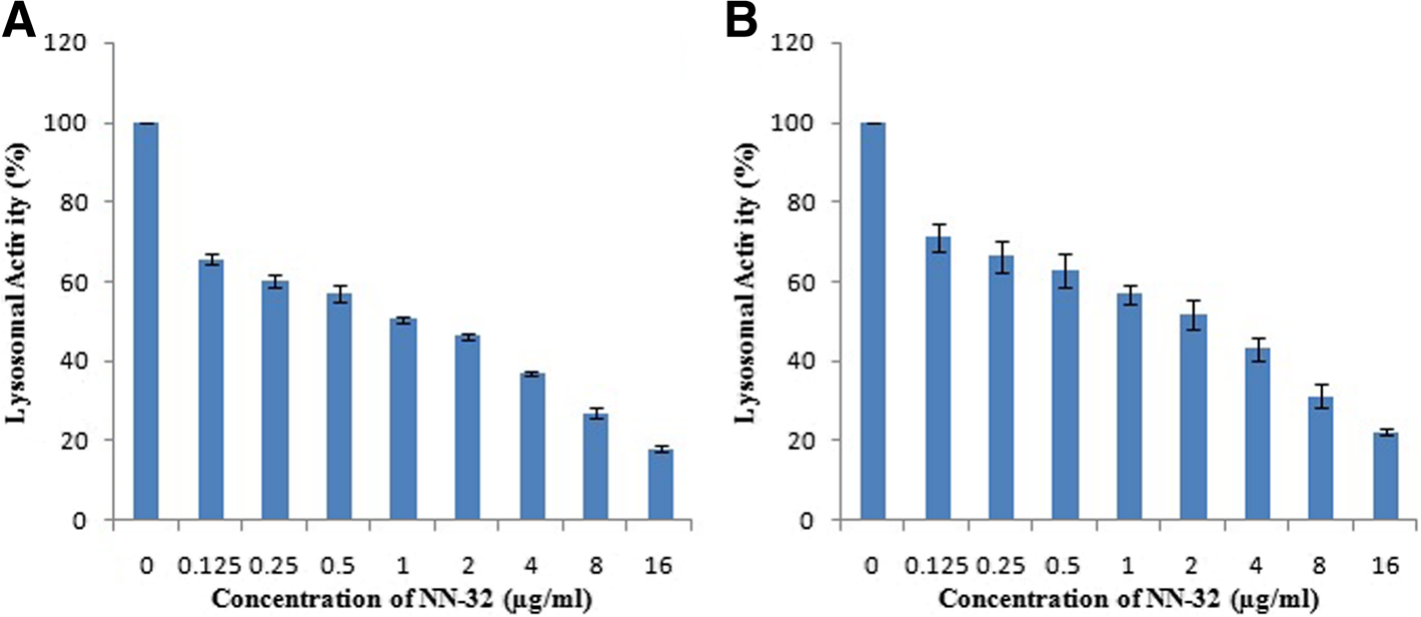
Lysosomal activity of breast cancer cell lines treated with NN-32 toxin for 72 h, determined by neural red uptake assay. **a** MCF-7 cell line and, (**b**) MDA-MB-231 cell line. Values are expressed as mean ± Std. Dev (*n* = 3)

Neutral Red uptake assay was performed to determine the lysosomal activity of MCF-7 and MDA-MB-231 cells treated with the NN-32 toxin showed a significant decrease in lysosomal activity in a dose-dependent manner (Fig. 7). This NR uptake assay showed the lower sensitivity than that of MTT assay, this may be because of the lower number of lysosomes in the breast cancer cell lines. Neutral red is a positively charged dye that diffuses through the cellular membrane of viable cells and accumulates in the lysosomes, thus the intensity of its staining is directly proportional to a number of viable cells.

### LDH release assay

**Fig. 8:**
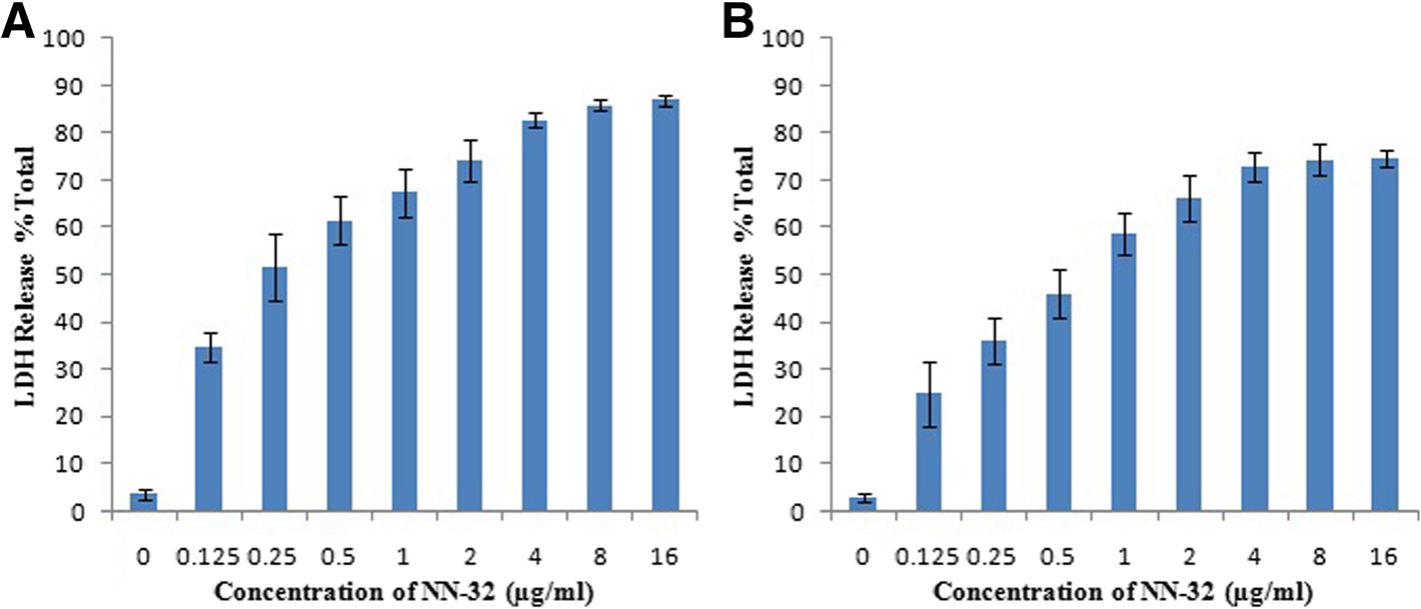
Lactate Dehydrogenase (LDH) release from (**a**) MCF-7 cells and, (**b**) MDA-MB-231 cells treated with the NN-32 toxin. Values are expressed as mean ± Std. Dev (*n* = 3)

LDH release in culture media is one of the indicators of cell death. Thus LDH release assay was performed to determine the cytotoxic activity of NN-32 toxin on human breast cancer cell lines. The LDH release curves of MCF-7 and MDA-MB-231 cell lines treated with different concentration of the NN-32 toxin showed that the cytotoxic effect of the NN-32 was concentration dependent (Fig. 8). The percent LDH release from MCF-7 cell lines after 72 h exposure to 0.25, 0.5, 1 and 2 μg/ml NN-32 toxin were 52, 61, 67 and 74% respectively. This effect is greater than those observed for the same concentration of NN-32 on MDA-MB-231 cell lines with 36, 46, 59 and 66% respectively after 72 h. However, a higher concentration of NN-32 toxin produced ever higher LDH release in both MCF-7 and MDA-MB-231 cells.

## Discussion

Since long, natural products from floral and faunal origin have been used for therapeutic purposes and around 87% of the human diseases are treated with these natural products and their related drugs [20].

Cardiotoxin-3 (CTX-3), a basic polypeptide of 60 amino acid residues from *Naja naja atra* venom induced apoptotic cell death accompanied by upgradation of both bax & endonuclease G and down regulation of bcl-x in K562 cells [21]. drCT-1 a heat stable, 7.2 kDa protein toxin from Indian Russell’s viper venom is supposed to possess anti-proliferative, cytotoxic and apoptotic activity on EAC mice and human leukemic cells (U937/K562) [22].

Salmosin, a disintegrin isolated from Korean snake venom, efficiently suppressed the growth of the metastatic tumor as well as the solid tumor in mice. Also, LAAO isolated from *Agkistrodon acutus* snake venom arrest tumor cells at a sub-G1 phase of cell cycle and induced apoptosis via fas pathway in A549 cells [23].

Crototoxin 2, a disintegrin isolated from *Crotalus atrox* induced cancer cell migration and lung tumor colonization in BALB/c mice [24]. VRCTC-310 is a natural product by combining two purified snake venom, a three protein fraction from *Crotalus durissus terrificus* venom and *Naja naja atra*, exerted an inhibitory effect on human and murine cell lines. In a phase-I study, 15 patients with refractory malignancies were given intramuscular injection daily for 30 days continuously to evaluate the tolerated dose (MTD), safety profile and pharmaceutical data. MCD was found out to be 0.017 mg/kg and recommended for phase-II studies [25]. Cytotoxin-II isolated from Caspian cobra (*Naja Naja oxiana*) showed potent anticancer effects in a breast carcinoma cell line via induction of apoptosis through lysosomal damage, production of intracellular ROS, mitochondrial damage and activation of caspases [26].

The present study showed that NN-32 isolated from *Naja naja* (Indian spectacled cobra) venom showed significant cytotoxicity against MCF-7 and MDA-MB-231 in both dose and time dependent manner, and considerably less towards normal breast cells (MCF-10A). The molecular mass of NN-32 was 6.7 kDa and N-terminal amino acid sequence for first 10 amino acid was LKCNKLVPLF [16].

MS/MS spectra of NN-32 searched against the NCBInr protein database using Mascot database search engine showed homology with Cytotoxin 2 (accession no. P01440), Cytotoxin 2a (accession no. P86538), Cytotoxin 3 (accession no. P24780) from *Naja naja*; Cytotoxin 1 (accession no. P01447), Cytotoxin 2 (accession no. P01445), Cytotoxin 3 (accession no. P01446) from *Naja kaouthia* and Cytotoxin 6 (accession no. P073858) from *Naja sputatrix*.

NKCT1 is a protein toxin isolated from *Naja Koauthia* venom after conjugation with gold nanoparticle i.e. GNP-NKCT1 showed anticancer effect both in vivo and in-vitro in EAC cells. GNP-NKCT1 induced caspase dependent apoptosis pathway in EAC cells and induced the late apoptotic stage and arrested cell cycle division at G0/G1 stage [27].

NN-32 showed antitumor and antioxidant properties against EAC bearing BALB/c mice [16]. NN-32 also showed anticancer activity in human leukemic U937 cells by promoting apoptosis, arresting cell cycle, suppressing vascular endothelial growth factor and matrix metalloproteinase activities [28].

The major anticancer mechanism activated by snake venom toxins is by mitochondria-dependent cell death pathway. Taiwan cobra cardiotoxin-III induced apoptosis by loss of mitochondrial membrane potential, release of cytochrome c, activation of caspase 9 and caspase 3, and altered expression of Bcl-2 family proteins [29, 30].

NN-32 showed cytotoxicity on EAC cells and it upregulated expression of proapoptotic proteins Bax and downregulated antiapoptotic protein Bcl-2 expression [16]. Increase in Bax: Bcl-2 ration results in series of events that lead to the conversion of procaspase 9 to active caspase 9. Caspase 9 is an effective downstream molecule of the mitochondrial pathway of apoptosis and will lead to the conversion of active caspase 3 from procaspase 3, which further trigger a cascade of intracellular events leading to programmed cell death [16]. Increase in expression of caspase 3 and caspase 9 in EAC cells after treatment suggested that NN-32 might have a role in the intrinsic pathway of apoptosis [16].

## Conclusion

The MTT, Antiproliferation, Neural red uptake and LDH release assay used to evaluate the cytotoxicity of NN-32 toxin purified from *Naja naja* venom on MCF-7 and MDA-MB-231 cell lines showed that the protein toxin NN-32 is significantly cytotoxic to these cell lines in a both dose and time dependent manner, and considerably less towards normal breast cells (MCF-10A). This finding highlights the potential of NN-32 toxin in the treatment of Breast cancer. However, detailed investigation of complete sequence, structure and molecular mechanism of action for this activity is required to potentiate the use this NN-32 toxin as a therapeutic agent for cancer treatment.

## Abbreviations

DMEM: Dulbecco’s modified eagle medium
DMSO: Dimethylsulphoxide
ER-: Estrogen receptor negative
ER +: Estrogen receptor positive
FBS: Fetal bovine serum
IC_50_: Half maximal inhibitory concentration
LDH: Lactate dehydrogenase
MS: Mass spectrometry
MTT: 3-(4,5-dimethylthiazol-2-Yl)-2,5-diphenyltetrazolium bromide
NR: Neutral red
